# Linkage, whole genome sequence, and biological data implicate variants in RAB10 in Alzheimer’s disease resilience

**DOI:** 10.1186/s13073-017-0486-1

**Published:** 2017-11-29

**Authors:** Perry G. Ridge, Celeste M. Karch, Simon Hsu, Ivan Arano, Craig C. Teerlink, Mark T. W. Ebbert, Josue D. Gonzalez Murcia, James M. Farnham, Anna R. Damato, Mariet Allen, Xue Wang, Oscar Harari, Victoria M. Fernandez, Rita Guerreiro, Jose Bras, John Hardy, Ronald Munger, Maria Norton, Celeste Sassi, Andrew Singleton, Steven G. Younkin, Dennis W. Dickson, Todd E. Golde, Nathan D. Price, Nilüfer Ertekin-Taner, Carlos Cruchaga, Alison M. Goate, Christopher Corcoran, JoAnn Tschanz, Lisa A. Cannon-Albright, John S. K. Kauwe, for the Alzheimer’s Disease Neuroimaging Initative

**Affiliations:** 1 Department of Biology, Brigham Young University, Provo, UT 84602 USA; 2 Washington University School of Medicine, St. Louis, MO 63110 USA; 3 Division of Genetic Epidemiology, Department of Internal Medicine, University of Utah School of Medicine, Salt Lake City, UT 84132 USA; 4 Present address: Department of Neuroscience, Mayo Clinic, 4500 San Pablo Road, Jacksonville, FL 32224 USA; 5 Department of Neuroscience, Mayo Clinic, Jacksonville, FL 32224 USA; 6 Department of Health Sciences Research, Mayo Clinic, Jacksonville, FL 32224 USA; 7 Department of Molecular Neuroscience, Institute of Neurology, University College London, London, UK; 8 Department of Nutrition, Dietetics, and Food Sciences, Utah State University, Logan, UT 84322 USA; 9 Department of Family Consumer & Human Development, Utah State University, Logan, UT 84322 USA; 10 National Institute on Aging, Bethesda, MD 21224 USA; 11 Center for Translational Research in Neurodegenerative Disease, McKnight Brain Institute, University of Florida, Department of Neuroscience, Gainesville, FL 32610 USA; 12 Institute for Systems Biology, 401 Terry Avenue N, Seattle, WA 98109 USA; 13 Departments of Neurology and Neuroscience, Mayo Clinic, Jacksonville, FL 32224 USA; 14 Departments of Neuroscience, Genetics and Genomic Sciences, and Neurology, Icahn School of Medicine at Mount Sinai, New York, NY 10029 USA; 15 Department of Mathematics and Statistics, Utah State University, Logan, UT 84322 USA; 16 Department of Psychology, Utah State University, Logan, UT 84322 USA; 17 George E. Wahlen Department of Veterans Affairs Medical Center, Salt Lake City, UT 84148 USA; 18 Departments of Biology and Neuroscience, Brigham Young University, Provo, UT 84602 USA

**Keywords:** Alzheimer’s disease, Protective variants, Whole genome sequencing, Utah Population Database, Linkage analyses

## Abstract

**Background:**

While age and the *APOE ε4* allele are major risk factors for Alzheimer’s disease (AD), a small percentage of individuals with these risk factors exhibit AD resilience by living well beyond 75 years of age without any clinical symptoms of cognitive decline.

**Methods:**

We used over 200 “AD resilient” individuals and an innovative, pedigree-based approach to identify genetic variants that segregate with AD resilience. First, we performed linkage analyses in pedigrees with resilient individuals and a statistical excess of AD deaths. Second, we used whole genome sequences to identify candidate SNPs in significant linkage regions. Third, we replicated SNPs from the linkage peaks that reduced risk for AD in an independent dataset and in a gene-based test. Finally, we experimentally characterized replicated SNPs.

**Results:**

Rs142787485 in *RAB10* confers significant protection against AD (*p* value = 0.0184, odds ratio = 0.5853). Moreover, we replicated this association in an independent series of unrelated individuals (*p* value = 0.028, odds ratio = 0.69) and used a gene-based test to confirm a role for *RAB10* variants in modifying AD risk (*p* value = 0.002). Experimentally, we demonstrated that knockdown of *RAB10* resulted in a significant decrease in Aβ42 (*p* value = 0.0003) and in the Aβ42/Aβ40 ratio (*p* value = 0.0001) in neuroblastoma cells. We also found that *RAB10* expression is significantly elevated in human AD brains (*p* value = 0.04).

**Conclusions:**

Our results suggest that *RAB10* could be a promising therapeutic target for AD prevention. In addition, our gene discovery approach can be expanded and adapted to other phenotypes, thus serving as a model for future efforts to identify rare variants for AD and other complex human diseases.

**Electronic supplementary material:**

The online version of this article (doi:10.1186/s13073-017-0486-1) contains supplementary material, which is available to authorized users.

## Background

A majority of Alzheimer’s disease (AD) genetic discoveries have been made using cutting edge study designs and large international collaborations [1–5]. However, despite these successes, the genetics of AD are still largely unsolved: 1) the majority of genetic variance is not explained by known AD markers [6]; 2) known AD markers are not helpful for predicting or diagnosing disease [7]; 3) a majority of remaining AD variants are likely to be rare [6, 8]; 4) and the functional consequences of known AD markers, or surrounding genetic variants, are unknown. These observations demonstrate the complexity of AD genetics and underscore the importance of developing new and targeted study designs capable of identifying rare genetic variants.

Recently, several possibly functional, rare variants with large protective [9, 10] and risk effects [11–13] have been identified for AD in *APP*, *APOE*, *PLD3*, and *TREM2* using novel study designs. The *TREM2* variant R47H, for example, was discovered using a study design that preserved statistical power by focusing solely on genetic variants that were likely to affect protein function [11, 12], whereas the *PLD3* variant, V232M, was identified using a family-based study design [13]. Identifying functional variants, such as the variants in *APP*, *APOE, PLD3*, and *TREM2* provide key insights into disease mechanisms [14, 15]. Since functional variants are more likely to represent tractable drug targets than other types of variants, they should be a major focus of AD genetics research [16, 17].

We report the development and use of an innovative, powerful approach to identify functional variants that provide AD resilience to high-risk individuals. First, we identified pedigrees *with a statistical excess of AD mortality* that also include at least four AD high-risk resilient individuals. Next, we performed linkage analysis in these families and used whole genome sequence (WGS) data from resilient individuals to interrogate identified linkage regions for candidate variants. We found promising variants in *RAB10* and *SAR1A*. Our *RAB10* findings were replicated in two independent series of unrelated individuals and in a gene-based test. Both *RAB10* and *SAR1A* are differentially expressed in human AD brains. Finally, we tested *RAB10* and *SAR1A* for biological impact in vitro. Our results suggest that *RAB10* variants impact risk for AD and that *RAB10* may represent a promising therapeutic target for AD prevention. In addition, our approach can be expanded and adapted to other phenotypes and serves as a model for future efforts to identify rare functional variants for AD and other complex human diseases.

## Methods

**Fig. 1:**
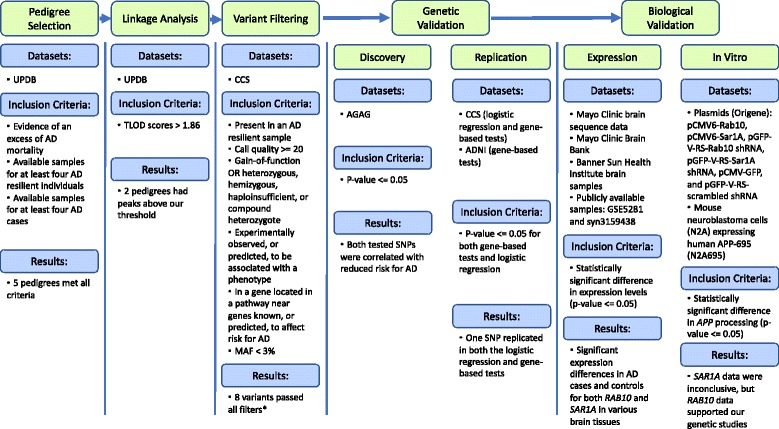
Overview of the process followed for analysis of data in this project. *TLOD* theta LOD, *UPDB* Utah Population Database, *CCS* Cache County Study on Memory Health and Aging, *AGAG* Alzheimer’s Genetics Analysis Group, *ADNI* Alzheimer’s Disease Neuroimaging Initiative. *Although eight variants passed all filters, two representative variants (one from each pedigree) were chosen based on known biology of the genes they reside in

We focused on understanding the underlying biology that protects certain high-risk individuals against AD. We term these individuals “AD resilient individuals” and define them as individuals who are at least 75 years old, cognitively normal, and carry at least one *APOE* ε*4* allele. Our approach consists of three key parts: linkage analysis and fine mapping, genetic analyses, and experimental biological validations. For simplicity, an overview of each step, datasets used, specific criteria applied, and high level results are presented in Fig. 1.

### Pedigree selection

We used the Utah Population Database (UPDB) to identify large pedigrees with an evidence of excess AD mortality (i.e., families with a higher number of AD deaths than expected). The UPDB is a population-based resource linking the computerized genealogy of the Utah pioneers, and their descendants, to various electronic health data repositories for the state, including Utah death certificates [18]. The UPDB includes over seven million individuals, 2.5 million of which have at least three generations of genealogical data and are descendants of the original founders of Utah; over one million of these individuals have at least 12 of their 14 immediate ancestors in the database.

Since 1904, Utah death certificates have been coded and linked to individuals in the UPDB, allowing us to identify all individuals where AD is included as a cause-of-death. AD as a specific cause-of-death was first introduced to the International Classification of Disease (ICD) in revision 9 and retained in revision 10. Deaths were only considered an AD death if the death certificate included AD ICD codes (ICD9 331.0; ICD10 F00 or G30) as a primary or contributing cause-of-death. This study used a uniform, consistent source for all diagnoses (AD that contributed to cause of death as evidenced by presence on a death certificate) and is not limited by bias introduced by study designs with inconsistent methods of diagnoses, or family recall of disease symptoms. The most significant limitation of this analysis is that coding for AD diagnosis has been present since 1979 (ICD versions 9 and 10). Given the breadth of our data, this limits our ability to identify cases that might be related across multiple generations (e.g., great grandparent/great grandchild), but our requirement for three generations of genealogy means that very distant relationships within the same generation are possible (Additional file 1: Figures S1 and S2). The most likely misclassification is that a death certificate for an individual who died with AD did not include AD as a cause of death. This would result in an underestimate of the number of AD deaths in a pedigree. Although individuals dying from AD may have been censored from our observation in this resource, the assumption can be made that cases are uniformly censored within cohorts across the resource, leading to conservative, but unbiased, estimates of relative AD mortality within pedigrees.

We used a method previously described by Kauwe et al. [19] to identify large pedigrees with a statistical excess of AD mortality. Briefly, each pedigree in the UPDB consists of all descendants of a set of UPDB founders. We identified pedigrees with an excess of AD deaths by comparing the observed (i.e., number of affected individuals in the pedigree) to the expected numbers of AD-affected individuals within the pedigree. The expected number of AD deaths was estimated using population-based, cohort-specific rates of AD death estimated from all Utah death certificates for individuals in the UPDB genealogy. To calculate the expected number of AD-affected individuals in a pedigree, first we divided all individuals in UPDB into cohorts based on birth year (5-year blocks), sex, and birth state (Utah or somewhere else), and normalized expected AD incidence to adjust for cohort-specific variation in death certificate information. All individuals were assigned to one of the resulting 132 cohorts. The proportion of individuals with AD in a cohort is the cohort-specific AD death rate for the UPDB genealogy population. This approach controls for differences in diagnosis and use of ICD codes for AD over time and space.

Next, we assessed each pedigree individually. To calculate the expected number of AD-affected individuals in a pedigree, we divided all pedigree descendants into cohorts, as described above, and multiplied the number of total descendants from the pedigree within the cohort by the cohort-specific AD rate previously calculated (i.e., proportion of AD individuals in the cohort) and summed the values across all cohorts within the pedigree. Therefore, the expected number of AD-affected individuals in a pedigree is the sum of the expected number of AD-affected individuals from each cohort in the pedigree. Finally, the observed number of AD descendants for a pedigree is calculated by counting individuals in the pedigree with an ICD code that indicates AD as a cause-of-death.

We estimated the relative risk (RR) for AD for each pedigree as the observed number of AD-affected descendants divided by the expected number of AD descendants. One-sided probabilities for the alternative hypothesis testing an RR > 1.0 were calculated under the null hypothesis RR = 1.0, with the assumption that the number of observed cases follows a Poisson distribution (an approximation to a sum of multiple binomial distributions representing the number of expected cases per cohort) with mean equal to the expected number of cases. This Poisson approximation is statistically appropriate for both rare and common phenotypes, being more conservative for a common disease. Pedigrees exhibiting excess AD descendants over expected were defined as high-risk.

### Samples

DNA and clinical phenotype data for AD cases and AD resilient samples for the linkage analysis were obtained from the Cache County Study on Memory Health and Aging (CCS), which has been described in more detail previously [20]. Briefly, the CCS was initiated in 1994 to investigate the association of *APOE* genotype and environmental exposures on cognitive function and dementia. This cohort of 5092 Cache County, Utah, residents (90% of those aged 65 years or older in 1994), has been followed continuously for over 15 years, with four triennial waves of data collection and additional clinical assessments for those at high-risk for dementia. DNA samples were obtained from 97.6% of participants. The Cache County population is exceptionally long-lived and ranked number one in life expectancy among all counties in the 1990 US Census [21]. All but one of the members of the CCS have been linked to the UPDB and their extended genealogies are known. This population was the source of most of the Centre d’Etude du Polymorphisme Humain (CEPH) families that have been used to represent Caucasians in many genetic studies worldwide, including the HapMap project. Recent analyses confirm that these data are representative of the general European-American population [22]. For this study, we needed both AD cases and resilient individuals identified in the same pedigrees.

First, we identified 232 AD resilient individuals (defined as those over age 75, cognitively healthy, and carrying at least one *APOE ε4* allele) from the CCS with a strong family history of AD. The set consists of 135 females and 97 males, with mean age of 81 years. As previously mentioned, each of these individuals carries at least one *APOE ε4* allele, and nine were homozygous for *APOE ε4*. We obtained WGS for 212 of these CCS samples using the Illumina HiSeq sequencer to an average depth of 40× and mapped the resulting reads with the Burrows-Wheeler Aligner (BWA) [23]. We performed variant calling using the Genome Analysis Toolkit (GATK) best practices (i.e., HaplotypeCaller) [24, 25]. We also genotyped each sample using the Illumina 2.5 M SNP Array for quality control and for use in linkage analyses.

Next, we identified 581 AD cases from the CCS, 492 of whom were followed from diagnosis to death. Since 2002, CCS participants with incident dementia have been followed prospectively in the Cache County Dementia Progression Study. An expert panel of neurologists, neuropsychologists, neuropsychiatrists, and a cognitive neuroscientist assigned final diagnoses of dementia following standard research protocols (e.g., NINCDS-ADRDA criteria for AD [20] or NINCDS-AIREN criteria for vascular dementia [26]). Each case was genotyped for the variants of interest using Taqman assays.

ADNI data used in the preparation of this article were obtained from the ADNI database (http://adni.loni.usc.edu/). The ADNI was launched in 2003 as a public–private partnership, led by Principal Investigator Michael W. Weiner, MD. The primary goal of ADNI has been to test whether serial magnetic resonance imaging (MRI), positron emission tomography (PET), other biological markers, and clinical and neuropsychological assessment can be combined to measure the progression of mild cognitive impairment (MCI) and early Alzheimer’s disease (AD). For up-to-date information, see http://www.adni-info.org/.

### Linkage analysis

Linkage analyses were conducted using pedigrees that included at least four AD resilient individuals and four AD cases. To identify key regions associated with AD resilience, we identified shared chromosomal segments among our AD resilient samples within each pedigree using MCLINK [27]. The set of OmniExpress SNPs considered were reduced to a set of high-heterozygosity markers with low or no pairwise linkage disequilibrium to enable unbiased linkage analysis. Pedigrees were analyzed using a general dominant model that assumed a disease gene frequency of 0.005 with penetrance estimates for carriers and non-carriers of 0.5 and 0.0005, respectively, and we considered different modes of inheritance and corrected for multiple tests [28]. We extracted inheritance information for each pedigree by reconstructing haplotypes using a Monte Carlo Markov Chain methodology with blocked Gibbs sampling [27–29]. For parametric analyses, MCLINK calculates robust multi-point linkage scores (theta LODs or TLODs) [29]. We consider TLOD scores > 1.86 (corresponding to a false-positive rate of one per genome) as suggestive evidence for linkage, and scores > 3.30 as significant, as defined by Lander and Kruglyak [30]. Using a conservative cutoff further allowed us to explore biological evidence for the maximal number of genes and variants, which are few by nature for this type of study.

Once linkage evidence was established via these methods, we utilized all SNP markers in the region to provide fine mapping localization evidence. Linkage evidence from each pedigree was considered independently.

### WGS variant filtering

Variants within the one LOD interval of the maximum linkage score were analyzed using the Ingenuity Variant Analysis and Tute Genomics Analysis programs (https://www.qiagenbioinformatics.com/products/ingenuity-variant-analysis/). For the Ingenuity Variant Analysis we used version 3.0.20140422 with content versions as follows: Ingenuity Knowledge Base (Arrakis 140408.002), COSMIC (v68) [31], dbSNP (build 138 (08/09/2013)), 1000 Genome Frequency (v3) [32], TargetScan (v6.2) [33], EVS (ESP6500 0.0.21), JASPAR (10/12/2009) [34], PhyloP hg18 (11/2009), PhyloP hg19 (01/2009) [35], Vista Enhancer hg18 (10/27/2007), Vista Enhancer hg19 (12/26/2010) [36], CGI Genomes (11/2011), SIFT (01/2013) [37], BSIFT (01/2013), The Cancer Genome Atlas (09/05/2013), PolyPhen-2 (HumVar Training set 2011_12) [38], Clinvar (02/11/2014).

All variants from the linkage regions were filtered as follows (see Additional file 1: Supplementary Note 1 for the effect each filter had on the number of variants):

- Included variants that are shared by resilient samples
- Included variants with call quality at least 20.0 in AD cases or resilient samples, outside the top 0.2% of the most exonically variable 100-base-pair windows in healthy public genomes (based on the 1000 Genomes Project), and outside the top 1% of the most exonically variable genes in healthy public genomes (based on the 1000 Genomes Project)
- Excluded variants if the allele frequency was at least 3% in the 1000 Genomes Project, the public Complete Genomics genomes, or the NHLBI ESP exomes (http://evs.gs.washington.edu/EVS/).
- Included variants associated with gain-of-function, or were heterozygous, hemizygous, haploinsufficient, or compound heterozygous
- Included variants experimentally observed to be associated with a phenotype by any of the following criteria: 1) pathogenic, possibly pathogenic, established gain-of-function in the literature, or inferred activating mutations by Ingenuity; 2) predicted gain-of-function by BSIFT; 3) located in a known microRNA binding site, or frameshift, in-frame indel, stop loss, missense, and not predicted to be benign by SIFT, or disrupt a splice site up to two bases into an intron; 4) deleterious to a microRNA or structural variant; 5) located in a known promoter binding or enhancer site; 6) located in an evolutionarily conserved region, determined by a phyloP *p* value ≥ 0.01, or 7) in an untranslated region
- Included variants absent in AD cases in the pedigree and present in a gene within two protein interaction connections upstream, or one connection downstream, of genes that are known, or predicted, to affect susceptibility to late-onset familial or sporadic AD

### Genetic validation analyses

We used three independent datasets for genetic validation analyses. First, all SNPs that met the filtering criteria (described above) were evaluated in a set of samples with sequence data. Then, significant markers from those analyses were genotyped and assessed for association in samples from the CCS. Finally, WGS data from the ADNI were analyzed. Our initial validation analysis was conducted using data from an augmented version of the Alzheimer Genetic Analysis Group dataset [12]. These data consist of whole exome sequences (WES) and WGS for 427 AD cases and 798 elderly controls originating from the United Kingdom and North America. The assembly and use of this dataset have been described in several studies (e.g., [39]). Briefly, since our dataset consisted of a mix of exomes captured using different kits, and whole genome sequences, we employed a highly conservative approach to variant selection to increase our confidence that analyzed variants are true positives. We limited our dataset of variants to only those genomic regions we expected to have been sequenced in each of the exomes (based on capture probes used for exome library preparation) and whole genomes. Next, we compiled a list of all the variants present in at least a single sample. We examined each of the variants from the list of total variants in each sample, whether or not the variant was called by the Genome Analysis Toolkit (GATK) best practices, and reassigned the genotype for that variant according to the following criteria. (1) If the variant was called by the GATK and passed all quality filters recommended by the GATK, we used the GATK genotype. (2) If no variant was called at the genomic position in question, we returned to the raw VCF file and if there were reads containing the variant, but the variant was not called because of failing filters or because only a small number of reads contain the variant, we set the genotype to missing for the sample. (3) Finally, if all the reads at this position for the sample indicated reference alleles, we set the genotype to homozygous reference.

Variants that were significant in the first validation analyses were genotyped in 523 AD cases and 3560 controls from the CCS (after exclusion of samples that were included in the linkage analysis). WGS from 191 AD cases and 279 controls from ADNI were used to conduct gene-based tests for association. These samples are described in detail on the ADNI website (http://adni.loni.usc.edu/data-samples/genetic-data/wgs/). Finally, there were no variants in these genes passed quality control in the Alzheimer’s Disease Sequencing Project samples.

We performed association analyses, using PLINK [40], between AD status and the top SNP in each linkage region (based on Ingenuity analyses), using a logistic regression and controlling for age, sex, and site. Given the linkage results, all tests were conducted assuming we were searching for a SNP with a protective effect against AD. We tested a single SNP from the linkage region in each family. As such, the alpha for the single SNP analyzed in each family is 0.05. Next, we used the sequence kernel association test (SKAT)-O to perform gene-based association tests in the ADNI samples to test whether each gene was a potential AD resilience gene [41]. SKAT-O was designed to combine both a burden test and a non-burden sequence kernel association test. It maximizes power from both test types, where burden tests are more powerful when the majority of variants in a region are both causal and in the same direction, and SKAT is adapted to regions with largely non-causal variants or causal variant effects are in different directions [41]. Thus, SKAT-O is ideal when the percentage of causal variants and their directions within a region are not known beforehand.

### Gene expression studies

We examined levels of *RAB10* and *SAR1A* expression in the temporal cortex of 80 brains with neuropathologic diagnosis of AD vs. 76 elderly control brains which lacked any diagnosis of neurodegenerative diseases. These brains were part of the Mayo Clinic RNA sequencing (RNAseq) cohort, described previously [42]. All subjects underwent RNAseq using Illumina HiSeq 2000, 101-base-pair, paired-end sequencing at the Mayo Clinic Genomic Core Facility. All the AD and some of the control brains were from the Mayo Clinic Brain Bank; whereas other control brains were from the Banner Sun Health Institute. Following quality control, raw read counts normalized according to conditional quantile normalization (CQN) using the Bioconductor package were used in the analyses. For differential gene expression (DGE) comparing AD vs. controls using the “Simple Model”, multi-variable linear regression analyses were conducted in R, using CQN normalized gene expression measures and including age-at-death, gender, RNA integrity number (RIN), brain tissue source, and flowcell as biological and technical covariates. We also performed DGE including cell-specific gene levels as covariates in addition to all covariates in the “Simple Model”, using the expression levels for the five central nervous system (CNS)-specific genes as follows: *ENO2* for neurons, *GFAP* for astrocytes, *CD68* for microglia, *OLIG2* for oligodendrocytes, and *CD34* for endothelial cells. The rationale for the “Comprehensive Model” is to account for any CNS cell-population changes that occur due to disease pathology. Significance accounting for multiple testing was assigned using q values which are based on false discovery rates [43].

Additionally, *RAB10* and *SAR1A* expression levels were evaluated in publicly available datasets from human AD and age-matched control brains (GSE5281 and syn3159438). The GSE5281 dataset was obtained from laser microdissected neurons from AD and control brains [44]. The syn3159438 dataset was obtained from anterior prefrontal cortex (APC), superior temporal gyrus (STG), parahippocampal gyrus (PHG), and pars opercularis (PO) [45]. RNA expression values were log transformed to achieve a normal distribution. An analysis of covariance, including age and sex as covariates, was used to determine association with disease status as previously described [46, 47].

### Biological validation studies

To further investigate the connection between *RAB10* and *SAR1A* and AD risk, we assessed the impact of gene overexpression and silencing on *APP* and ß-amyloid levels in N2A695 cells.

We used the following plasmids for this study: pCMV6-Rab10 (Origene), pCMV6-Sar1A (Origene), pGFP-V-RS-Rab10 shRNA (Origene), pGFP-V-RS-Sar1A shRNA (Origene), pCMV-GFP, and pGFP-V-RS-scrambled shRNA (Origene). The optimal shRNA for each gene was selected from four possible shRNAs based on the plasmid producing the most robust knockdown in vitro.

Mouse neuroblastoma cells (N2A) expressing human APP-695 isoform (termed N2A695) were used in this study [48]. N2A695 cells were plated and grown in Dulbecco’s modified Eagle medium (DMEM) and Opti-MEM supplemented with 1% L-glutamine, 10% FBS and 1% antibiotic-anti-microbial solution, and 200 μg/mL G418. Upon reaching confluency, cells were transiently transfected using Lipofectamine 2000 (Life Technologies). Culture media was changed 24 h after transfection. After an additional 24 h, cell media and cell pellets were collected for subsequent analysis. Nine independent replicates were performed for each condition.

Cell death following overexpression and knockdown was assessed by measuring LDH release in the cellular medium (Thermo Scientific) according to the manufacturer’s instructions. Percentage of cytotoxicity was then calculated following manufacturer’s recommendations:

$$ \%\mathrm{Cytotoxicity}=\left(\left(\mathrm{Transfected}\;\mathrm{LDH}\hbox{--} \mathrm{Spontaneous}\;\mathrm{LDH}\right)\div \left(\mathrm{Maximum}\;\mathrm{LDH}\hbox{--} \mathrm{Spontaneous}\;\mathrm{LDH}\right)\right)\times 100 $$

To assess overexpression and silencing of *RAB10* and *SAR1A*, total RNA was isolated from N2A695 cells 48 h after transfection using RNeasy (Qiagen). RNA was converted to cDNA using the High-capacity cDNA reverse transcription kit (Thermo Fisher Scientific). Gene expression was analyzed by real-time PCR using an ABI-7900 real time PCR system. Taqman (Thermo Fisher Scientific) real time PCR assays were used to measure the expression of *RAB10* (Mm00489481_m1), *SAR1A* (Mm01150424_m1), and the housekeeping gene *GAPDH* (Hs02758991_g1). Samples were run in triplicate. To avoid amplification interference, expression assays were run in separate wells from the housekeeping gene.

Real-time data were analyzed using the comparative threshold cycle (C_T_) method [49]. Briefly, the C_T_ is the PCR cycle at which fluorescence rises above background, allowing us to calculate the original RNA levels. For the comparative C_T_ method, the average C_T_ for *RAB10* or *SAR1A* were normalized to the average C_T_ for *GAPDH*. The resulting value was then corrected for assay efficiency. Samples with a standard error of 20% or less were analyzed. *RAB10* shRNA resulted in a 54% reduction of endogenous *RAB10*, and *SAR1A* shRNA resulted in a 26% reduction of endogenous *SAR1A*.

To assess steady-state levels of *RAB10*, *SAR1A*, and *APP*, cell lysates were extracted in lysis buffer (50 mM Tris pH7.6, 1 mM EDTA, 150 mM NaCl, 1% TritonX-100, protease inhibitor cocktail) on ice. Lysates were centrifuged at 14,000xg for 10 minutes at 4 °C and the resulting supernatant was saved for SDS-PAGE and immunoblotting. Total protein concentration was measured by BCA assay according to manufacturer’s protocol (Thermo Scientific).

Standard sodium dodecyl sulfate-polyacrylamide gel electrophoresis (SDS-PAGE) was performed using 4–12% Criterion Tris-HCl gels (Bio-Rad). Samples were boiled in Laemmli sample buffer prior to electrophoresis [50]. Immunoblots were probed with 9E10 (myc; Sigma), 6E10 (APP, sAPP_α_; Covance), 22C11 (APP, sAPP_total_; Millipore), sAPPβ (Clontech), and CT695 (APP, CTF-β and CTF-ɑ; ThermoFisher).

The levels of human Aβ40 and Aβ42 were measured from conditioned cell culture media by sandwich ELISA as described by the manufacturer (Thermo Fisher Scientific). ELISA values were obtained (pg/mL) and corrected for total intracellular protein (μg/mL) based on BCA measurements from cell lysates.

Aβ concentrations are expressed as mean ± standard deviation obtained from at least three separate experiments in each group. Data were assessed by one-way analysis of variance (ANOVA). When ANOVA indicated significant differences, the Student’s *t*-test was used with Bonferroni correction for multiple comparisons. Results presented are representative and those with *p* values < 0.05 were considered significant.

## Results

### Pedigree selection and linkage analysis

We identified five pedigrees that passed all filtering criteria: 1) evidence of an excess of AD deaths; 2) available samples for at least four AD resilient individuals (i.e., elderly *APOE ε4* carriers); and 3) available samples for at least four AD cases. Two pedigrees reached our 1.86 TLOD cutoff for linkage analysis (Additional file 1: Figures S1 and S2).

In the first pedigree (Additional file 1: Figure S1), we detected a linkage region with a TLOD score of 2.21 on chromosome 2. This peak is located between rs4341893 and rs2252032 (chr2:20935817-36872196; 2p23-22), and includes 14,898 SNPs and 101 genes. In the second pedigree (Additional file 1: Figure S2), we detected evidence of linkage with a TLOD score of 2.10 in two adjacent regions on chromosome 10, which includes 10,686 variants in 138 genes. These peaks are located between rs10823229 and rs7900882, and rs7918631 and rs3740382, respectively, and hereafter are treated as a single peak (chr10:68572823-103419457; 10q22.1-24.3). We failed to detect evidence of linkage in the three other pedigrees.

### Association with AD risk

We extracted all variants from whole genomes in the two linkage regions. We identified eight candidate variants that passed all filters (Table 1; Additional file 1: Supplementary Note 1), and selected one candidate SNP from each of the two peaks for further analysis. Each of these variants, in *RAB10* (rs142787485) and *SAR1A* (rs7653), respectively, had statistically significant associations with AD in the Alzheimer’s Genetic Analysis Group. We deliberately selected our candidate SNPs from *RAB10* and *SAR1A* because these genes interact with *APP* [51, 52]. We observed significant associations in the Alzheimer’s Genetic Analysis Group in the protective direction for both SNPs (rs142787485, *RAB10*, *p* value = 0.018, odds ratio (OR) = 0.58; rs7653, *SAR1A*, *p* value = 0.0049, OR = 0.35). Both SNPs are rare, with 1000 Genomes minor allele frequencies of 0.0136 and 0.0168, for rs142787485 and rs7653, respectively.

**Table 1:** Variants in the linkage region after filtering

SNP	Chromosome	Position	Gene	Variant type	MAF (controls/cases)	MAF (1000 Genomes/ExAC
rs142787485	2	26358156	RAB10	3′ UTR	0.041/0.028	0.0152/NA
rs77747916	2	29405333	CLIP4	3′ UTR	0.0099/0.0069	0.0087/NA
rs41291171	2	33623713	LTBP1	3′ UTR	0.049/0.049	0.0243/NA
rs7653	10	71910316	SAR1A	3′ UTR	0.03/0.0092	0.0175/NA
rs143318821	10	102105884	SCD	Promoter	NA	0.004/NA
rs41562219	10	103340081	POLL	Exonic p.D337D; ncRNA p.D429D	0.0075/0.0080	0.0029/0.0034
chr10:103347900	10	103347900	POLL; DPCD	Promoter; 5′UTR	0.0012/0	NA/NA
rs116928523	10	103912209	NOLC1	Exonic p.L14L	0.0075/0.0057	0.003/0.006

For each variant, the dbSNP identifier (*SNP*), chromosome, position, closest gene(s) (*Gene*), variant type, minor allele frequency (*MAF*) in controls and cases, and frequency from the 1000 Genomes Project and Exome Aggregation Consortium (ExAC) are provided (NA if not present in the database)

Given significant findings in the sequence data, we genotyped both rs142787485 (*RAB10*) and rs7653 (*SAR1A*) in samples from the Cache County Study on Memory Health and Aging (CCS), an independent dataset of 544 cases and 3605 controls. While odds ratios for both markers were in the predicted protective direction (Table 2), we detected significant association with rs142787485 (*p* value = 0.028, OR = 0.69), but not rs7653 (*p* value = 0.26, OR = 0.87). Gene-based tests conducted in the CCS and Alzheimer’s Disease Neuroimaging Initiative (ADNI) samples using SKAT-O resulted in a significant association for *RAB10* (*p* value = 0.002), but not *SAR1A* (*p* value = 1.00).

**Table 2:** Replication test results in the CCS for selected SNPs

SNP	Gene	*p* value	OR (95% CI)	MAF controls/Cases
Rs142787485	*RAB10*	0.028	0.69 (0.47–0.99)	0.045/0.031
Rs7653	*SAR1A*	0.26	0.87 (0.54–1.31)	0.025/0.021

For each variant the dbSNP identifier (*SNP*), closest gene, *p* value and odds ratio (*OR*) in the CCS, and minor allele frequency (*MAF*) in controls and cases are shown

### Differential expression of *RAB10* and *SAR1A* in AD brains

To determine whether *RAB10* and *SAR1A* expression are altered in AD brains, we examined transcriptomic data from 80 AD brains and 76 age-matched control brains (Mayo Clinic Dataset). *RAB10* mRNA levels were significantly higher (Table 3) in the temporal cortex of AD brains compared to controls. To replicate our *RAB10* findings, we analyzed a publicly available dataset containing 260 brains from AD cases and age-matched controls from the Mount Sinai Brain Bank (syn3159438). We observed a significant increase in *RAB10* expression in AD brains (STG *p* value = 0.0285) and a marginal association between *RAB10* expression and plaque load (STG *p* value = 0.0579). AD brains are characterized by extensive neuronal loss. To evaluate whether the effect on *RAB10* expression in AD brains is driven by altered cell composition within the brain homogenates, we analyzed *RAB10* expression after correcting for cell composition in the Mayo Clinic Dataset (Comprehensive Model). After correction for cell composition, *RAB10* expression levels remained significantly elevated in the temporal cortex of AD brains (Table 3). We replicated this finding by examining *RAB10* expression in neurons isolated from AD brains (GSE5281). We found that *RAB10* expression was higher in AD neurons compared with controls (*p* value = 0.0456).

**Table 3:** Mayo Clinic Brain RNAseq data for SAR1A and RAB10 genes in AD vs. control brains

Gene ID	Chr	Start	Stop	Coding length	Tissue	Model	Comparison	Dx.Beta	EffectDirection	Dx.SE	Dx.pValue	Dx.qValue
*RAB10*	chr2	26256976	26360323	3631	TCX	Compre	Control_vs_AD	0.14	UpInAD	0.04	1.55E-03	7.12E-02
*RAB10*	chr2	26256976	26360323	3631	TCX	Simple	Control_vs_AD	0.32	UpInAD	0.05	6.96E-09	1.50E-06
*SAR1A*	chr10	71907045	71930279	6914	TCX	Compre	Control_vs_AD	-0.12	DownInAD	0.05	1.27E-02	1.80E-01
*SAR1A*	chr10	71907045	71930279	6914	TCX	Simple	Control_vs_AD	-0.10	DownInAD	0.04	2.07E-02	5.12E-02

*Gene ID*, ENSEMBL gene ID; *Tissue*, *TCX* temporal cortex; *Dx.Beta*, coefficient of effect in AD in comparison to controls; *Dx.SE*, standard error of effect; *Dx.pValue*, significance of effect (uncorrected); *Dx.qValue*, significance corrected using FDR-based q-values

We found that *SAR1A* expression was significantly reduced in AD brains compared with age-matched controls (APC *p* value = 0.04; STG *p* value = 0.0005; PO *p* value = 0.0000279) and associated with plaque load (APC *p* value = 0.062; STG *p* value = 0.0005; PG *p* value = 0.00638; PO *p* value = 0.00000911). This association was validated in human neurons from AD cases and controls, where *SAR1A* levels were significantly lower in AD neurons compared with age-matched controls (*p* value = 0.0008). We observed a trend towards lower *SAR1A* levels in AD brains in the Mayo Clinic Dataset; however, *SAR1A* levels were not significantly different in the temporal cortex between AD cases and controls (Table 3).

### Over-expression and knockdown of *RAB10* and *SAR1A*

**Fig. 2:**
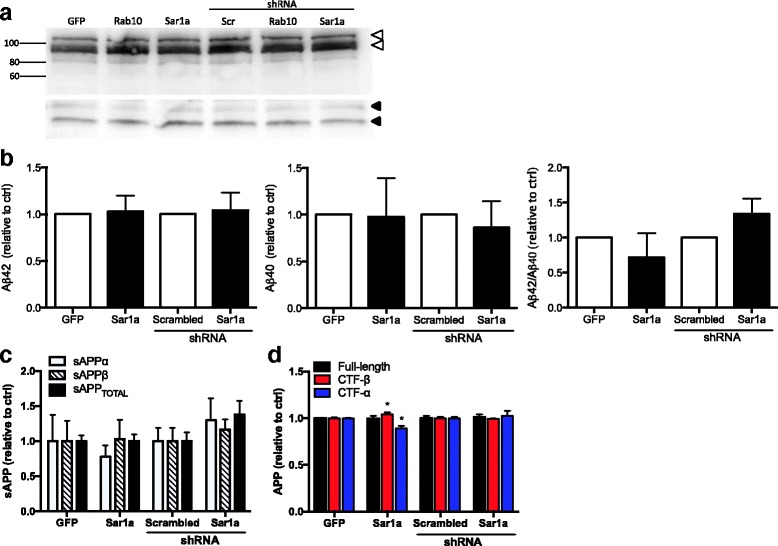
*Sar1a* subtly affects APP processing in vitro. **a** Full-length APP levels are not altered by Sar1a expression. Immunoblots of N2A695 cells transiently transfected with vectors expressing GFP, Sar1a, scrambled shRNA, or shRNA specific to Sar1a. *Open arrowhead*, *APP*; *closed arrowhead*, CTF-β and CTF-ɑ. **b** Sar1a expression does not significantly alter extracellular amyloid-beta levels. Conditioned media from N2A695 cells overexpressing or silencing Sar1a were analyzed by ELISA and resulting values were expressed relative to control. **c** *Sar1a* expression does not significantly alter sAPP levels. Quantification of immunoblots of sAPPalpha, sAPPbeta, and sAPPtotal. **d** *Sar1a* overexpression alters CTF-β and CTF-ɑ. Quantification of immunoblots of full-length *APP*, CTF-β, and CTF-ɑ. Graphs represent mean ± SEM from at least three independent experiments. *p value < 0.05

**Fig. 3:**
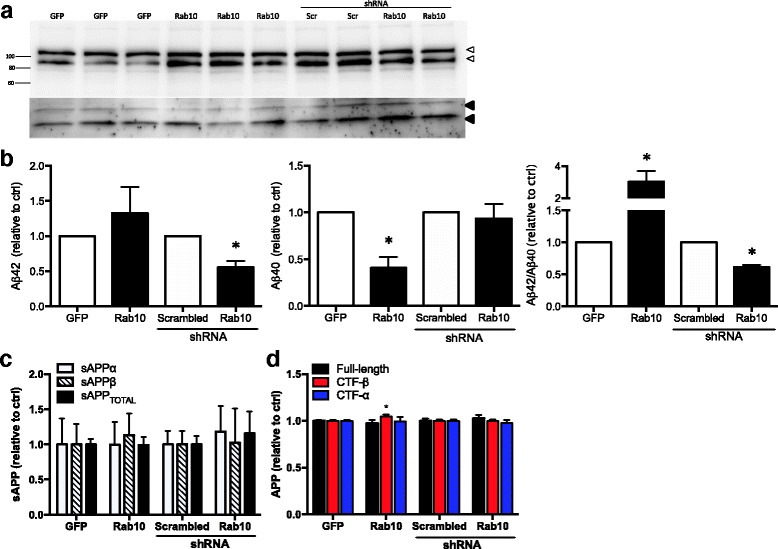
*Rab10* alters APP processing in vitro*.* **a** Full-length *APP* levels are not altered by *Rab10* expression. Immunoblots of N2A695 cells transiently transfected with vectors expressing GFP, *Rab10*, scrambled shRNA, or shRNA specific to *Rab10. Open arrowheads*, *APP*; *closed arrowheads*, CTF-β and CTF-ɑ. **b** Rab10 expression alters extracellular amyloid-beta levels. Conditioned media from N2A695 cells overexpressing or silencing *Rab10* were analyzed by ELISA and resulting values were expressed relative to control. **c** Rab10 expression does not significantly alter sAPP levels. Quantification of immunoblots of sAPPalpha, sAPPbeta, and sAPPtotal. **d** *Rab10* overexpression alters CTF-β. Quantification of immunoblots of full-length *APP*, CTF-β, and CTF-ɑ. Graphs represent mean ± SEM from at least three independent experiments. *p value < 0.05

To examine previous reports of biochemical interactions between *RAB10* and APP and between *SAR1A* and *APP*, we examined the effects of overexpressing and silencing *RAB10* and *SAR1A* on *APP* processing in mouse neuroblastoma cells [51, 52]. Overexpression and silencing of *SAR1A* and *RAB10* did not affect cell viability. *SAR1A* overexpression and modest silencing of *SAR1A* expression failed to produce a significant change in full-length intracellular *APP*, sAPP levels, or in extracellular Aβ levels (Fig. 2). Interestingly, overexpression of *SAR1A* produced an increase in CTF-β and corresponding decrease in CTF-ɑ relative to GFP-only (*p* value = 0.0010 and 0.0382, respectively). Overexpressing *RAB10* resulted in a significant increase in the Aβ42/Aβ40 ratio (*p* value = 0.0133) and CTF-β (*p* value = 0.0409), while knockdown of endogenous *RAB10* resulted in a significant decrease in Aβ42 (*p* value = 0.0003) and in the Aβ42/Aβ40 ratio (*p* value = 0.0001) (Fig. 3b; Table 4). Aβ levels were altered in the absence of an accompanying change in full-length, intracellular *APP*, or sAPP levels (Fig. 3a, c; Table 4).

**Table 4:** Impact of RAB10 over-expression and knockdown in N2A695 cells

		*p* value	Fold change (± SEM)
RAB10 over-expression	Aβ40	0.0011	−0.5933 ± 0.1162
	Aβ42	0.2008	0.3249 ± 0.3709
	Aβ42/Aβ40	0.0133	2.014 ± 0.6894
	sAPPalpha	0.3436	0.02252 ± 0.05198
	sAPPbeta	0.0187	0.1466 ± 0.04776
	sAPPtotal	0.3581	−0.01433 ± 0.03673
RAB10 knockdown	Aβ40	0.4095	−0.06635 ± 0.2825
	Aβ42	0.0003	−0.4453 ± 0.08428
	Aβ42/Aβ40	0.0001	−0.3876 ± 0.02243
	sAPPalpha	0.2899	0.1131 ± 0.188
	sAPPbeta	0.2025	0.2457 ± 0.2642
	sAPPtotal	0.3233	0.1446 ± 0.2922

The *p* value for the comparison to GFP control (over-expression) and scrambled shRNA (knockdown) and fold change for each amyloid measurement are provided

## Discussion

We exploited strengths in the Utah Population Database (UPDB) and CCS to identify five pedigrees with a statistical excess of AD deaths. Using linkage analysis, we identified linkages in two pedigrees on chromosomes 2 and 10. The linkage region on chromosome 2 is far (>90 Mb) from known AD genome-wide association study (GWAS) genes, and no known AD GWAS genes are on chromosome 10.

Multiple lines of evidence support a role for *RAB10* in AD. We detected evidence for linkage in *RAB10*, significant associations in the Alzheimer’s Genetic Analysis Group (*p* value = 0.0184), replication in an independent set of samples from the CCS (*p* value = 0.028), and replication by gene-based tests in WGS data from ADNI (*p* value = 0.002). Furthermore, we assessed the effect of *RAB10* expression on Aβ. Approximately 50% knockdown of *RAB10* resulted in a 45% reduction in Aβ42 levels (*p* value = 0.0003) and a 61% reduction in the Aβ42/Aβ40 (*p* value = 0.0001) ratio. These findings are consistent with previous reports that *RAB10* silencing affects Aβ levels [52] and extend those findings by defining the effects of *RAB10* overexpression and silencing on APP processing, including Aβ isoforms, APP-CTF, and sAPP. Based on our results, we hypothesize that Rab10 impacts APP processing through direct interaction with APP [51]. The relationship between *RAB10* and Aβ suggests *RAB10* may affect γ-secretase-mediated cleavage of *APP*, and the secretion and degradation of cleaved Aβ. Furthermore, *RAB10* is expressed in all cell types in human and mouse brains [53, 54], trends toward increased expression in neurons isolated from AD brains [55], and has higher brain expression levels in AD cases than controls. *RAB10* plays a role in endocytosis, which has been implicated in AD [56, 57], and is involved in membrane trafficking regulation and moving proteins from the Golgi apparatus to the membrane [58, 59]. It also has a role in neurotransmitter release, phagosome maturation, and *GLUT4* translocation [57]. In neurons, *RAB10* is involved in axonogenesis through the regulation of vesicular membrane trafficking toward the axonal plasma membrane [60]. Our experimental results and previous reports support our genetic discovery. These functional findings are consistent with the identification of a rare variant in *RAB10* that is over-represented in cognitively normal, elderly individuals. Adding further interest to this discovery, these individuals have high genetic risk for AD, yet remain healthy. Thus, targeting *RAB10* could represent a novel therapeutic strategy for treating AD.

The variant in *SAR1A* did not replicate in an independent set of samples from the CCS, but the effect was in the expected direction (odds ratio = 0.87, 95% confidence interval (CI) 0.54–1.31). The exact function of *SAR1A*, a GTPase, is unknown, but it is believed to be involved in membrane trafficking and is part of the endoplasmic reticulum to Golgi apparatus transport complex [61]. We tested the effect of *SAR1A* overexpression and knockdown on Aβ levels and our functional data were inconclusive. We achieved only modest silencing of *SAR1A* expression. This also contributes to the inconclusive nature of our results. Yet, additional evidence supports a possible role for *SAR1A* in AD. *SAR1A* binds *APP* [51] and is widely expressed in all regions of both the human and mouse brains [53, 54], and *SAR1A* expression is lower in neurons isolated from AD brains compared to controls [55]. Rs7653 is located in the 3′ untranslated region of *SAR1A* and could possibly be involved in regulation of translation by modifying microRNA binding, but no definitive data on functional impact is available and no clear bioinformatic predictions can be made at this time. To date, rs7653 is not associated with any phenotypes in the NHGRI-EBI GWAS Catalog (accessed 18 September 2017) [62].

In summary, we used an innovative approach to identify rare variants that affect risk for AD. Our approach provides several advantages compared to other study designs. First, these large and wide pedigrees capture even distantly related individuals and therefore provide many informative meioses. Second, each pedigree has a significant excess of AD mortality over multiple generations and distant relationships when compared to general Utah rates, thus provides sets of distantly related individuals who likely have a strong genetic component to their AD, which narrows the likely genomic location to a small window. Third, since we have a set of healthy, high-risk, elderly individuals, some of which are members of families with an excess of AD deaths, these individuals likely share protective genetics and this study design is ideal to identify protective genetic variants.

Despite the advantages of this approach, there are several limitations to the design. First, the nature of the pedigree selection and rarity of the AD resilient samples led to sampling that made obtaining significant LOD scores very difficult. As a result, we obtained suggestive LOD scores in two of the five pedigrees, but no significant LOD scores in any of the pedigrees. However, any concerns about genetic results should be at least somewhat alleviated by the experimental evidence supporting the genetic discoveries.

Second, in tests for AD excess in the UPDB, we identified affected individuals based on presence of International Classification of Disease (ICD)9 or ICD10 codes for AD on a Utah death certificate. Assignment of cause-of-death from death certificates is recognized to be imprecise. Due to the challenge of diagnosing AD, especially in the past, it is much more likely that AD as cause-of-death is missing from death certificates where it belongs, as compared to having been incorrectly included. This makes our estimates of AD death rates extra conservative, and any biases that exist, exist across all UPDB data equally.

Third, genealogy data used to define relationships might have included some relationships that were not biological and some relationship data might have been censored due to failure to link records. Some results may require validation in other populations and results based only on Utah data can only be extended to similar populations of European descent. Despite these potential limitations in our genetics work, our biological findings suggest that *RAB10* may regulate Aβ levels, thus altering risk for AD.

## Conclusions

Using an innovative study design and unique resources, we have obtained evidence that rare variation in *RAB10* may provide resilience to AD. Linkage and sequence analyses, replication using both SNP and gene-based tests, and in vitro functional work suggest that *RAB10* may represent effective targets for AD prevention and therapy. Finally, we have provided a model for an effective research design for studying complex traits.

## Additional file

Additional file 1: Supplementary Note 1. Variant filtration process. **Figures S1 and S2** The pedigrees for RAB10 and SAR1A. (PDF 815 kb)

## Abbreviations

(SKAT)-O: Sequence kernel association test
AD: Alzheimer’s disease
ADNI: Alzheimer’s Disease Neuroimaging Initiative
APC: Anterior prefrontal cortex
CCS: Cache County Study on Memory Health and Aging
CEPH: Centre d’Etude du Polymorphisme Humain
CQN: Conditional quantile normalization
DGE: Differential gene expression
GATK: Genome Analysis Toolkit
ICD: International Classification of Disease
PHG: Parahippocampal gyrus
PO: Pars opercularis
RIN: RNA integrity number
RR: Relative risk
STG: Superior temporal gyrus
UPDB: Utah Population Database
WES: Whole exome sequence
WGS: Whole genome sequence

## References

[1] Harold D, Abraham R, Hollingworth P, Sims R, Gerrish A, Hamshere ML, Pahwa JS, Moskvina V, Dowzell K, Williams A (2009). Genome-wide association study identifies variants at CLU and PICALM associated with Alzheimer’s disease. Nat Genet.

[2] Lambert JC, Heath S, Even G, Campion D, Sleegers K, Hiltunen M, Combarros O, Zelenika D, Bullido MJ, Tavernier B (2009). Genome-wide association study identifies variants at CLU and CR1 associated with Alzheimer’s disease. Nat Genet.

[3] Lambert JC, Ibrahim-Verbaas CA, Harold D, Naj AC, Sims R, Bellenguez C, DeStafano AL, Bis JC, Beecham GW, Grenier-Boley B (2013). Meta-analysis of 74,046 individuals identifies 11 new susceptibility loci for Alzheimer’s disease. Nat Genet.

[4] Hollingworth P, Harold D, Sims R, Gerrish A, Lambert JC, Carrasquillo MM, Abraham R, Hamshere ML, Pahwa JS, Moskvina V (2011). Common variants at ABCA7, MS4A6A/MS4A4E, EPHA1, CD33 and CD2AP are associated with Alzheimer’s disease. Nat Genet.

[5] Naj AC, Jun G, Beecham GW, Wang LS, Vardarajan BN, Buros J, Gallins PJ, Buxbaum JD, Jarvik GP, Crane PK (2011). Common variants at MS4A4/MS4A6E, CD2AP, CD33 and EPHA1 are associated with late-onset Alzheimer’s disease. Nat Genet.

[6] Ridge PG, Hoyt KB, Boehme K, Mukherjee S, Crane PK, Haines JL, Mayeux R, Farrer LA, Pericak-Vance MA, Schellenberg GD (2016). Assessment of the genetic variance of late-onset Alzheimer’s disease. Neurobiol Aging.

[7] Ebbert MT, Ridge PG, Wilson AR, Sharp AR, Bailey M, Norton MC, Tschanz JT, Munger RG, Corcoran CD, Kauwe JS (2014). Population-based analysis of Alzheimer’s disease risk alleles implicates genetic interactions. Biol Psychiatry.

[8] Ridge PG, Mukherjee S, Crane PK, Kauwe JS, Alzheimer’s Disease Genetics C (2013). Alzheimer’s disease: analyzing the missing heritability. PLoS One.

[9] Jonsson T, Atwal JK, Steinberg S, Snaedal J, Jonsson PV, Bjornsson S, Stefansson H, Sulem P, Gudbjartsson D, Maloney J (2012). A mutation in APP protects against Alzheimer’s disease and age-related cognitive decline. Nature.

[10] Medway CW, Abdul-Hay S, Mims T, Ma L, Bisceglio G, Zou F, Pankratz S, Sando SB, Aasly JO, Barcikowska M, Siuda J, Wszolek ZK, Ross OA, Carrasquillo M, Dickson DW, Graff-Radford N, Petersen RC, Ertekin-Taner N, Morgan K, Bu G1, Younkin SG. ApoE variant p.V236E is associated with markedly reduced risk of Alzheimer’s disease. Mol Neurodegener. 2014;9:11. doi: 10.1186/1750-1326-9-11. 10.1186/1750-1326-9-11 PMC3995879 24607147

[11] Jonsson T, Stefansson H, Ph DS, Jonsdottir I, Jonsson PV, Snaedal J, Bjornsson S, Huttenlocher J, Levey AI, Lah JJ, et al. Variant of TREM2 Associated with the Risk of Alzheimer’s Disease. N Engl J Med. 2013:368(2);107-16. doi: 10.1056/NEJMoa1211103. Epub 2012 Nov 14. 10.1056/NEJMoa1211103 PMC3677583 23150908

[12] Guerreiro R, Wojtas A, Bras J, Carrasquillo M, Rogaeva E, Majounie E, Cruchaga C, Sassi C, Kauwe JS, Younkin S (2013). TREM2 variants in Alzheimer’s disease. N Engl J Med.

[13] Cruchaga C, Karch CM, Jin SC, Benitez BA, Cai Y, Guerreiro R, Harari O, Norton J, Budde J, Bertelsen S (2014). Rare coding variants in the phospholipase D3 gene confer risk for Alzheimer’s disease. Nature.

[14] Bettens K, Sleegers K, Van Broeckhoven C (2013). Genetic insights in Alzheimer’s disease. Lancet Neurol.

[15] Karch CM, Goate AM (2015). Alzheimer’s disease risk genes and mechanisms of disease pathogenesis. Biol Psychiatry.

[16] Ross CJ, Liu G, Kuivenhoven JA, Twisk J, Rip J, van Dop W, Excoffon KJ, Lewis SM, Kastelein JJ, Hayden MR (2005). Complete rescue of lipoprotein lipase-deficient mice by somatic gene transfer of the naturally occurring LPLS447X beneficial mutation. Arterioscler Thromb Vasc Biol.

[17] Rader DJ (2005). Gain-of-function mutations and therapeutic implications: lipoprotein lipase S447X to the rescue. Arterioscler Thromb Vasc Biol.

[18] Skolnick M, Cairns J, Lyons J, Skolnick M (1980). The Utah Geneological database: a resource for genetic epidemiology. Banbury Report No 4: Cancer incidence in defined populations.

[19] Kauwe JS, Ridge PG, Foster NL, Cannon-Albright LA (2013). Strong evidence for a genetic contribution to late-onset Alzheimer’s disease mortality: a population-based study. PLoS One.

[20] Breitner JC, Wyse BW, Anthony JC, Welsh-Bohmer KA, Steffens DC, Norton MC, Tschanz JT, Plassman BL, Meyer MR, Skoog I (1999). APOE-epsilon4 count predicts age when prevalence of AD increases, then declines: the Cache County Study. Neurology.

[21] Murray C (1998). U.S. Patterns of mortality by county and race: 1965-1994.

[22] Sharp AR, Ridge PG, Bailey MH, Boehme KL, Norton MC, Tschanz JT, Munger RG, Corcoran CD, Kauwe JS, Alzheimer’s Disease Neuroimaging I (2014). Population substructure in Cache County, Utah: the Cache County study. BMC Bioinformatics.

[23] Li H, Durbin R (2009). Fast and accurate short read alignment with Burrows-Wheeler transform. Bioinformatics.

[24] McKenna A, Hanna M, Banks E, Sivachenko A, Cibulskis K, Kernytsky A, Garimella K, Altshuler D, Gabriel S, Daly M (2010). The Genome Analysis Toolkit: a MapReduce framework for analyzing next-generation DNA sequencing data. Genome Res.

[25] Li H, Handsaker B, Wysoker A, Fennell T, Ruan J, Homer N, Marth G, Abecasis G, Durbin R, Genome Project Data Processing S (2009). The Sequence Alignment/Map format and SAMtools. Bioinformatics.

[26] Roman GC, Tatemichi TK, Erkinjuntti T, Cummings JL, Masdeu JC, Garcia JH, Amaducci L, Orgogozo JM, Brun A, Hofman A (1993). Vascular dementia: diagnostic criteria for research studies. Report of the NINDS-AIREN International Workshop. Neurology.

[27] Thomas A, Gutin A, Abkevich V, Bansal A (2000). Multilocus linkage analysis by blocked Gibbs sampling. Stat Comput.

[28] Camp NJ, Farnham JM (2001). Correcting for multiple analyses in genomewide linkage studies. Ann Hum Genet.

[29] Abkevich V, Camp NJ, Gutin A, Farnham JM, Cannon-Albright L, Thomas A (2001). A robust multipoint linkage statistic (tlod) for mapping complex trait loci. Genet Epidemiol..

[30] Lander E, Kruglyak L (1995). Genetic dissection of complex traits: guidelines for interpreting and reporting linkage results. Nat Genet.

[31] Forbes SA, Beare D, Gunasekaran P, Leung K, Bindal N, Boutselakis H, Ding M, Bamford S, Cole C, Ward S (2015). COSMIC: exploring the world’s knowledge of somatic mutations in human cancer. Nucleic Acids Res.

[32] Genomes Project C, Auton A, Brooks LD, Durbin RM, Garrison EP, Kang HM, Korbel JO, Marchini JL, McCarthy S, McVean GA (2015). A global reference for human genetic variation. Nature.

[33] Agarwal V, Bell GW, Nam JW, Bartel DP. Predicting effective microRNA target sites in mammalian mRNAs. Elife. 2015;4. doi: 10.7554/eLife.05005. 10.7554/eLife.05005 PMC4532895 26267216

[34] Mathelier A, Fornes O, Arenillas DJ, Chen CY, Denay G, Lee J, Shi W, Shyr C, Tan G, Worsley-Hunt R (2016). JASPAR 2016: a major expansion and update of the open-access database of transcription factor binding profiles. Nucleic Acids Res.

[35] Pollard KS, Hubisz MJ, Rosenbloom KR, Siepel A (2010). Detection of nonneutral substitution rates on mammalian phylogenies. Genome Res.

[36] Visel A, Minovitsky S, Dubchak I, Pennacchio LA (2007). VISTA Enhancer Browser--a database of tissue-specific human enhancers. Nucleic Acids Res.

[37] Kumar P, Henikoff S, Ng PC (2009). Predicting the effects of coding non-synonymous variants on protein function using the SIFT algorithm. Nat Protoc.

[38] Adzhubei IA, Schmidt S, Peshkin L, Ramensky VE, Gerasimova A, Bork P, Kondrashov AS, Sunyaev SR (2010). A method and server for predicting damaging missense mutations. Nat Methods.

[39] Sassi C, Ridge PG, Nalls MA, Gibbs R, Ding J, Lupton MK, Troakes C, Lunnon K, Al-Sarraj S, Brown KS (2016). Influence of coding variability in APP-Abeta metabolism genes in sporadic Alzheimer’s disease. PLoS One.

[40] Purcell S, Neale B, Todd-Brown K, Thomas L, Ferreira MA, Bender D, Maller J, Sklar P, de Bakker PI, Daly MJ (2007). PLINK: a tool set for whole-genome association and population-based linkage analyses. Am J Hum Genet.

[41] Lee S, Emond MJ, Bamshad MJ, Barnes KC, Rieder MJ, Nickerson DA, Christiani DC, Wurfel MM, Lin X, Team NGESP-ELP (2012). Optimal unified approach for rare-variant association testing with application to small-sample case-control whole-exome sequencing studies. Am J Hum Genet.

[42] Allen M, Carrasquillo MM, Funk C, Heavner BD, Zou F, Younkin CS, Burgess JD, Chai HS, Crook J, Eddy JA (2016). Human whole genome genotype and transcriptome data for Alzheimer’s and other neurodegenerative diseases. Sci Data..

[43] Reiner A, Yekutieli D, Benjamini Y (2003). Identifying differentially expressed genes using false discovery rate controlling procedures. Bioinformatics.

[44] Liang WS, Reiman EM, Valla J, Dunckley T, Beach TG, Grover A, Niedzielko TL, Schneider LE, Mastroeni D, Caselli R (2008). Alzheimer’s disease is associated with reduced expression of energy metabolism genes in posterior cingulate neurons. Proc Natl Acad Sci U S A.

[45] Zhang B, Gaiteri C, Bodea LG, Wang Z, McElwee J, Podtelezhnikov AA, Zhang C, Xie T, Tran L, Dobrin R (2013). Integrated systems approach identifies genetic nodes and networks in late-onset Alzheimer’s disease. Cell.

[46] Karch CM, Ezerskiy LA, Bertelsen S, Goate AM, Alzheimer’s Disease Genetics C (2016). Alzheimer’s disease risk polymorphisms regulate gene expression in the ZCWPW1 and the CELF1 loci. PLoS One.

[47] Karch CM, Jeng AT, Nowotny P, Cady J, Cruchaga C, Goate AM (2012). Expression of novel Alzheimer’s disease risk genes in control and Alzheimer’s disease brains. PLoS One.

[48] Thinakaran G, Teplow DB, Siman R, Greenberg B, Sisodia SS (1996). Metabolism of the “Swedish” amyloid precursor protein variant in neuro2a (N2a) cells. Evidence that cleavage at the “beta-secretase” site occurs in the golgi apparatus. J Biol Chem.

[49] Muller PY, Janovjak H, Miserez AR, Dobbie Z. Processing of gene expression data generated by quantitative real-time RT-PCR. Biotechniques. 2002;32(6):1372–1374, 1376, 1378–1379. 12074169

[50] Laemmli UK (1970). Cleavage of structural proteins during the assembly of the head of bacteriophage T4. Nature.

[51] Olah J, Vincze O, Virok D, Simon D, Bozso Z, Tokesi N, Horvath I, Hlavanda E, Kovacs J, Magyar A (2011). Interactions of pathological hallmark proteins: tubulin polymerization promoting protein/p25, beta-amyloid, and alpha-synuclein. J Biol Chem.

[52] Udayar V, Buggia-Prevot V, Guerreiro RL, Siegel G, Rambabu N, Soohoo AL, Ponnusamy M, Siegenthaler B, Bali J, Aesg (2013). A paired RNAi and RabGAP overexpression screen identifies Rab11 as a regulator of beta-amyloid production. Cell Rep.

[53] Hawrylycz MJ, Lein ES, Guillozet-Bongaarts AL, Shen EH, Ng L, Miller JA, van de Lagemaat LN, Smith KA, Ebbert A, Riley ZL (2012). An anatomically comprehensive atlas of the adult human brain transcriptome. Nature.

[54] Zhang Y, Chen K, Sloan SA, Bennett ML, Scholze AR, O’Keeffe S, Phatnani HP, Guarnieri P, Caneda C, Ruderisch N (2014). An RNA-sequencing transcriptome and splicing database of glia, neurons, and vascular cells of the cerebral cortex. J Neurosci.

[55] Liang WS, Dunckley T, Beach TG, Grover A, Mastroeni D, Ramsey K, Caselli RJ, Kukull WA, McKeel D, Morris JC (2008). Altered neuronal gene expression in brain regions differentially affected by Alzheimer’s disease: a reference data set. Physiol Genomics.

[56] Ginsberg SD, Alldred MJ, Counts SE, Cataldo AM, Neve RL, Jiang Y, Wuu J, Chao MV, Mufson EJ, Nixon RA (2010). Microarray analysis of hippocampal CA1 neurons implicates early endosomal dysfunction during Alzheimer’s disease progression. Biol Psychiatry.

[57] Mitra S, Cheng KW, Mills GB (2011). Rab GTPases implicated in inherited and acquired disorders. Semin Cell Dev Biol.

[58] Hutagalung AH, Novick PJ (2011). Role of Rab GTPases in membrane traffic and cell physiology. Physiol Rev.

[59] Bao S, Zhu J, Garvey WT (1998). Cloning of Rab GTPases expressed in human skeletal muscle: studies in insulin-resistant subjects. Horm Metab Res.

[60] English AR, Voeltz GK (2013). Rab10 GTPase regulates ER dynamics and morphology. Nat Cell Biol.

[61] Watson P, Townley AK, Koka P, Palmer KJ, Stephens DJ (2006). Sec16 defines endoplasmic reticulum exit sites and is required for secretory cargo export in mammalian cells. Traffic.

[62] Welter D, MacArthur J, Morales J, Burdett T, Hall P, Junkins H, Klemm A, Flicek P, Manolio T, Hindorff L (2014). The NHGRI GWAS Catalog, a curated resource of SNP-trait associations. Nucleic Acids Res.

