# Correction to: Linkage, whole genome sequence, and biological data implicate variants in RAB10 in Alzheimer’s disease resilience

**DOI:** 10.1186/s13073-018-0516-7

**Published:** 2018-01-12

**Authors:** Perry G. Ridge, Celeste M. Karch, Simon Hsu, Ivan Arano, Craig C. Teerlink, Mark T. W. Ebbert, Josue D. Gonzalez Murcia, James M. Farnham, Anna R. Damato, Mariet Allen, Xue Wang, Oscar Harari, Victoria M. Fernandez, Rita Guerreiro, Jose Bras, John Hardy, Ronald Munger, Maria Norton, Celeste Sassi, Andrew Singleton, Steven G. Younkin, Dennis W. Dickson, Todd E. Golde, Nathan D. Price, Nilüfer Ertekin-Taner, Carlos Cruchaga, Alison M. Goate, Christopher Corcoran, JoAnn Tschanz, Lisa A. Cannon-Albright, John S. K. Kauwe, for the Alzheimer’s Disease Neuroimaging Initiative

**Affiliations:** 1 Department of Biology, Brigham Young University, Provo, UT 84602 USA; 2 Washington University School of Medicine, St. Louis, MO 63110 USA; 3 Division of Genetic Epidemiology, Department of Internal Medicine, University of Utah School of Medicine, Salt Lake City, UT 84132 USA; 4 Present address: Department of Neuroscience, Mayo Clinic, 4500 San Pablo Road, Jacksonville, FL 32224 USA; 5 Department of Neuroscience, Mayo Clinic, Jacksonville, FL 32224 USA; 6 Department of Health Sciences Research, Mayo Clinic, Jacksonville, FL 32224 USA; 7 Department of Molecular Neuroscience, Institute of Neurology, University College London, London, UK; 8 Department of Nutrition, Dietetics, and Food Sciences, Utah State University, Logan, UT 84322 USA; 9 Department of Family Consumer & Human Development, Utah State University, Logan, UT 84322 USA; 10 National Institute on Aging, Bethesda, MD 21224 USA; 11 Center for Translational Research in Neurodegenerative Disease, McKnight Brain Institute, University of Florida, Department of Neuroscience, Gainesville, FL 32610 USA; 12 Institute for Systems Biology, 401 Terry Avenue N, Seattle, WA 98109 USA; 13 Departments of Neurology and Neuroscience, Mayo Clinic, Jacksonville, FL 32224 USA; 14 Departments of Neuroscience, Genetics and Genomic Sciences, and Neurology, Icahn School of Medicine at Mount Sinai, New York, NY 10029 USA; 15 Department of Mathematics and Statistics, Utah State University, Logan, UT 84322 USA; 16 Department of Psychology, Utah State University, Logan, UT 84322 USA; 17 George E. Wahlen Department of Veterans Affairs Medical Center, Salt Lake City, UT 84148 USA; 18 Departments of Biology and Neuroscience, Brigham Young University, Provo, UT 84602 USA

## Correction

The original version of this article [1] unfortunately contained a typographical error. The ‘Alzheimer’s Disease Neuroimaging Initiative’ was erroneously included as ‘Alzheimer’s Disease Neuroimaging Initative’ in the author list of the article.

Furthermore, the funders, Donor’s Cure and Chatbooks were inadvertently left out of the ‘Funding’ section. The revised ‘Funding’ section should read:

This work was supported by National Institute on Aging (R01AG042611 and RF1AG054052 to JSKK; R01AG032990, RFAG051504 to NET; U01AG046139 to NET, SGY, TEG, NDP; AG046374 to CMK; U01AG052411 to AMG; RO1AG18712 to RM, R01AG11380); National Institute of Neurological Disorders and Stroke (R01NS080820 to NET); Mayo Clinic Center for Individualized Medicine, Donor’s Cure, and Chatbooks. This work was supported by access to equipment made possible by the Hope Center for Neurological Disorders, and the Departments of Neurology and Psychiatry at Washington University School of Medicine.

The correct author group name has been included in the author list of this ‘Correction’ and revised in the original article. The omitted acknowledgement of the funders, Donor’s Cure and Chatbooks has also been included in the original article.
