# Supplementary material for: Linkage, whole genome sequence, and biological data implicate variants in RAB10 in Alzheimer’s disease resilience
Source: Genome Med. 2017 Nov 29;9:100. doi: 10.1186/s13073-017-0486-1 (PMC5706401; doi:10.1186/s13073-017-0486-1)
Supplement: Additional file 1: — Supplementary Note 1. Variant filtration process. Figures S1 and S2 The pedigrees for RAB10 and SAR1A. (PDF 815 kb) [file 13073_2017_486_MOESM1_ESM.pdf]

Supplementary Figures.

Supplementary Figure 1. Chromosome 2 pedigree diagram. Individuals with asterisks share a haplotype across SNPs within the peak linkage (TLOD = 2.02) region containing *RAB10* (Build 38, bp 20100662-42546485).

Supplementary Figure 2. Chromosome 10 pedigree diagram. Individuals with asterisks share a haplotype across SNPs within the peak linkage (TLOD = 2.03) region containing *SAR1A* (Build 38, bp 69011889-71240936).

Supplementary Figure 1

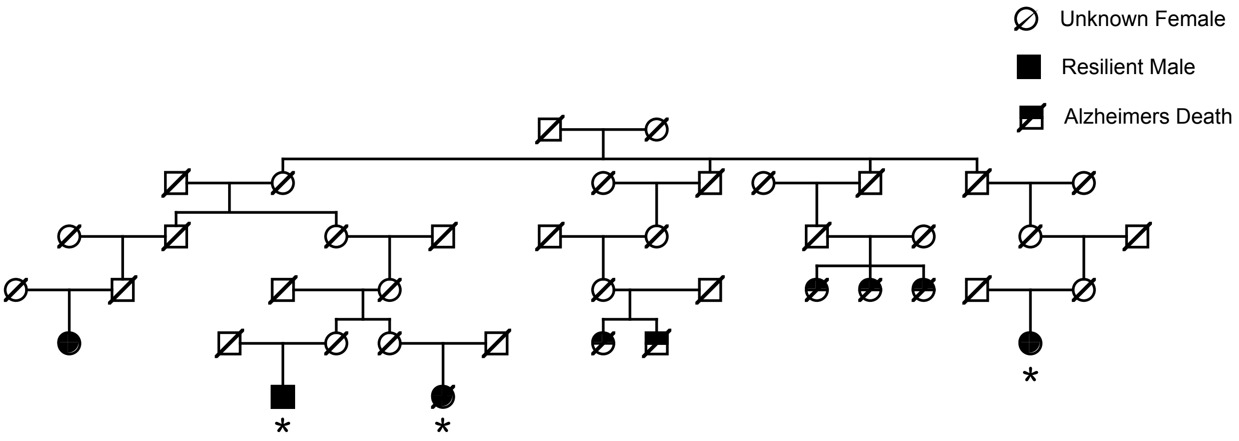

Supplementary Figure 2

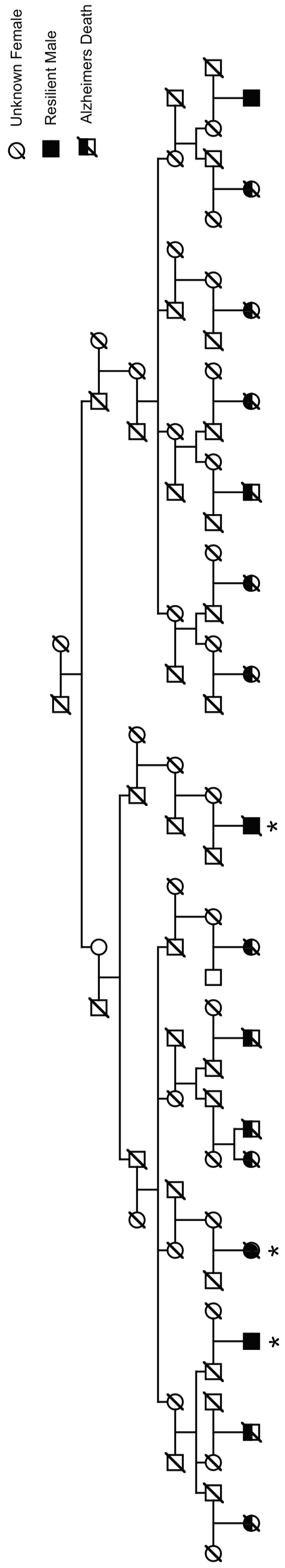

Supplementary Note 1

Variant Filtering Process and Results:

- Variants that are shared by resilient samples
  - 25,584 variants
- Call quality at least 20.0 in AD cases or resilient samples, outside the top 0.2% of the most exonically variable 100 base pair windows in healthy public genomes (based on the 1000 Genomes Project), and outside the top 1% of the most exonically variable genes in healthy public genomes (based on the 1000 Genomes Project)
  - 25,499 variants
- Variants were excluded if the allele frequency was at least 3% in the 1000 Genomes Project, the public Complete Genomics genomes, or the NHLBI ESP exomes (http://evs.gs.washington.edu/EVS/).
  - 795 variants
- Associated with gain-of-function, or were heterozygous, hemizygous, haploinsufficient, or compound heterozygous, and occur in at least one of the AD resilient samples
  - 265 variants
- Experimentally observed to be associated with a phenotype by any of the following criteria: 1) pathogenic, possibly pathogenic, established gain-of-function in the literature, or inferred activating mutations by Ingenuity, 2) predicted gain-of-function by BSIFT, 3) located in a known microRNA binding site, or frameshift, in-frame indel, stop loss, missense, and not predicted to be

benign by SIFT, or disrupt a splice site up to 2 bases into an intron, 4) deleterious to a microRNA or structural variant, 5) located in a known promoter binding or enhancer site, 6) located in an evolutionary-conserved region, determined by a phyloP p-value >= 0.01, or 7) in an untranslated region

  - 215 variants

- In a gene within two protein interaction connections upstream, or one connection downstream, of genes that are known, or predicted, to affect susceptibility to late-onset familial or sporadic AD
  - 8 variants
